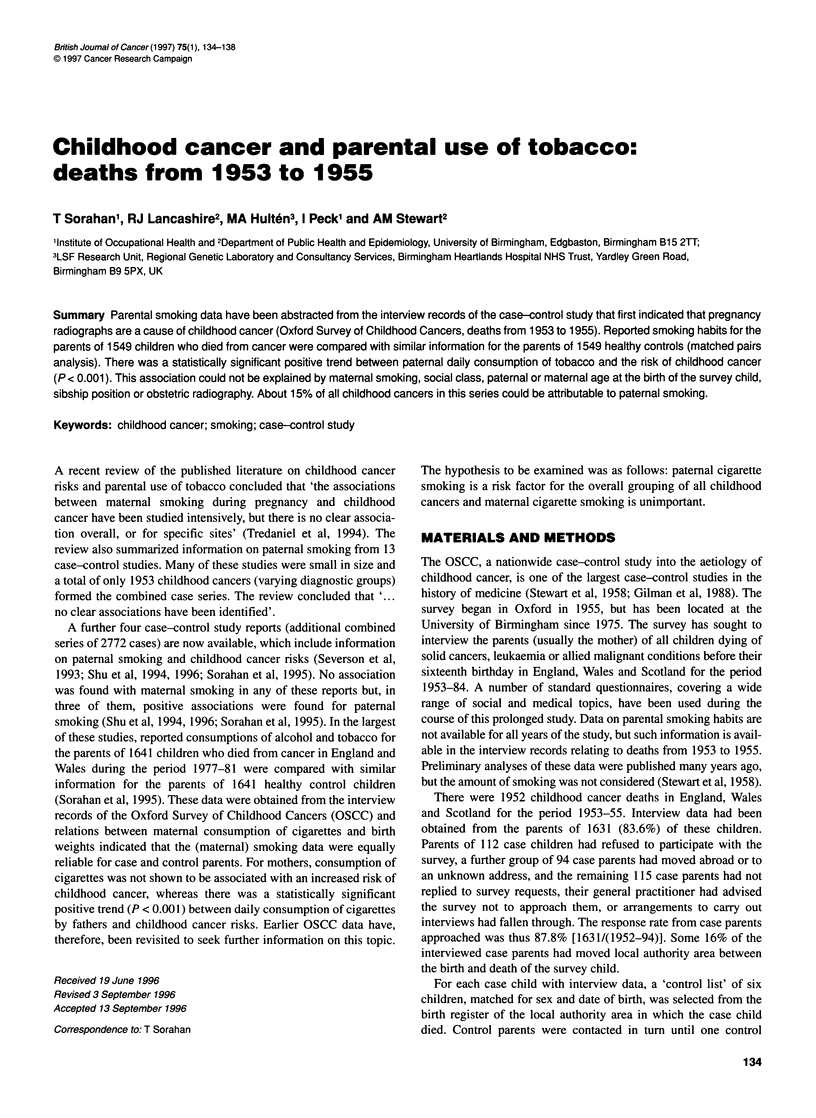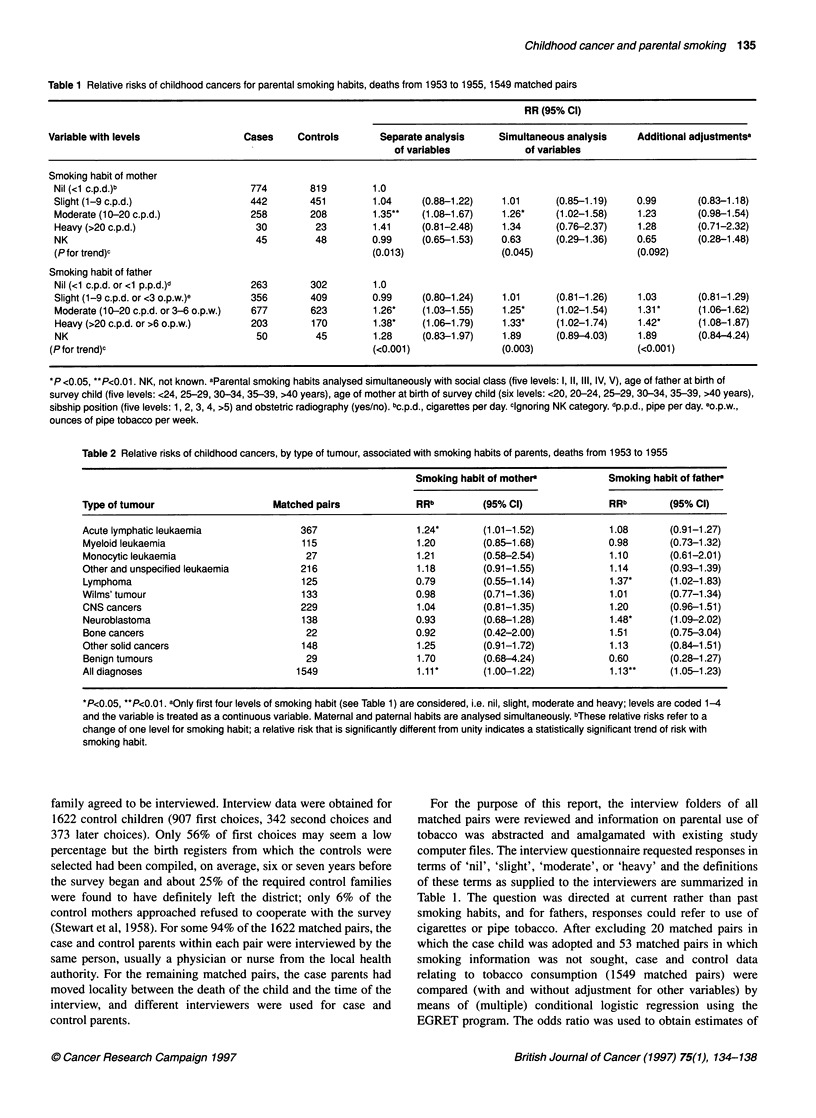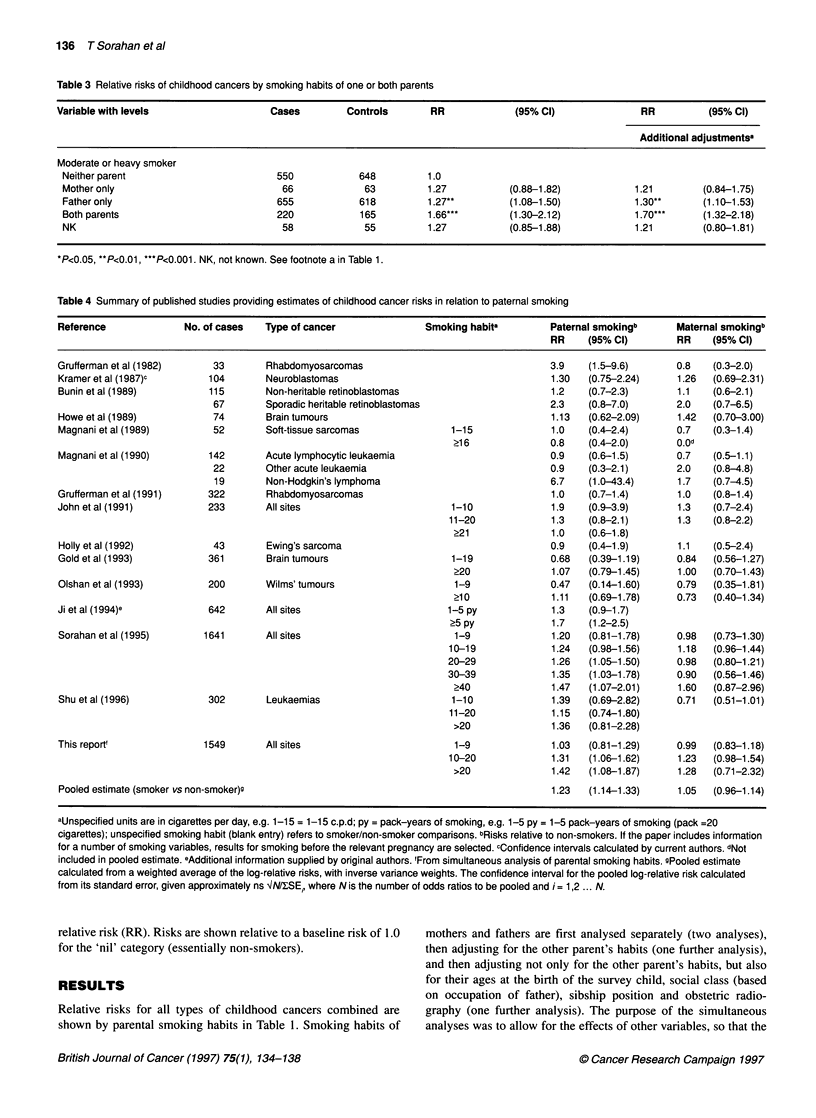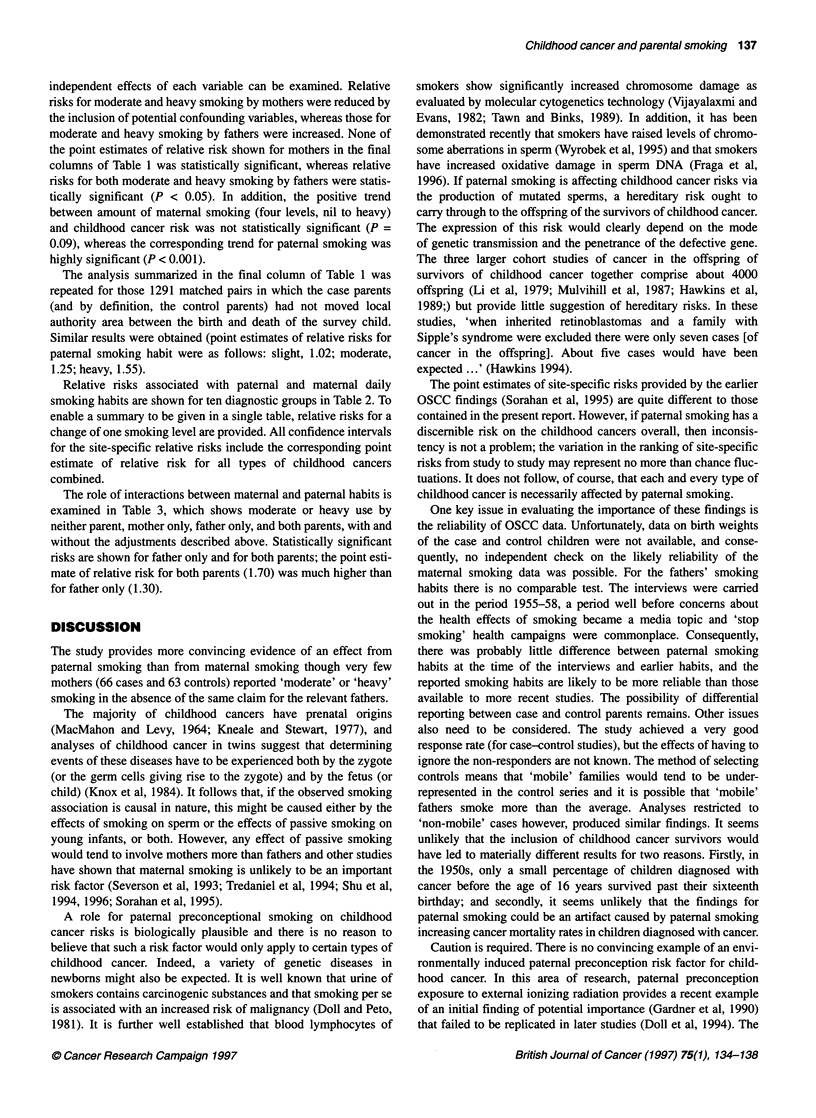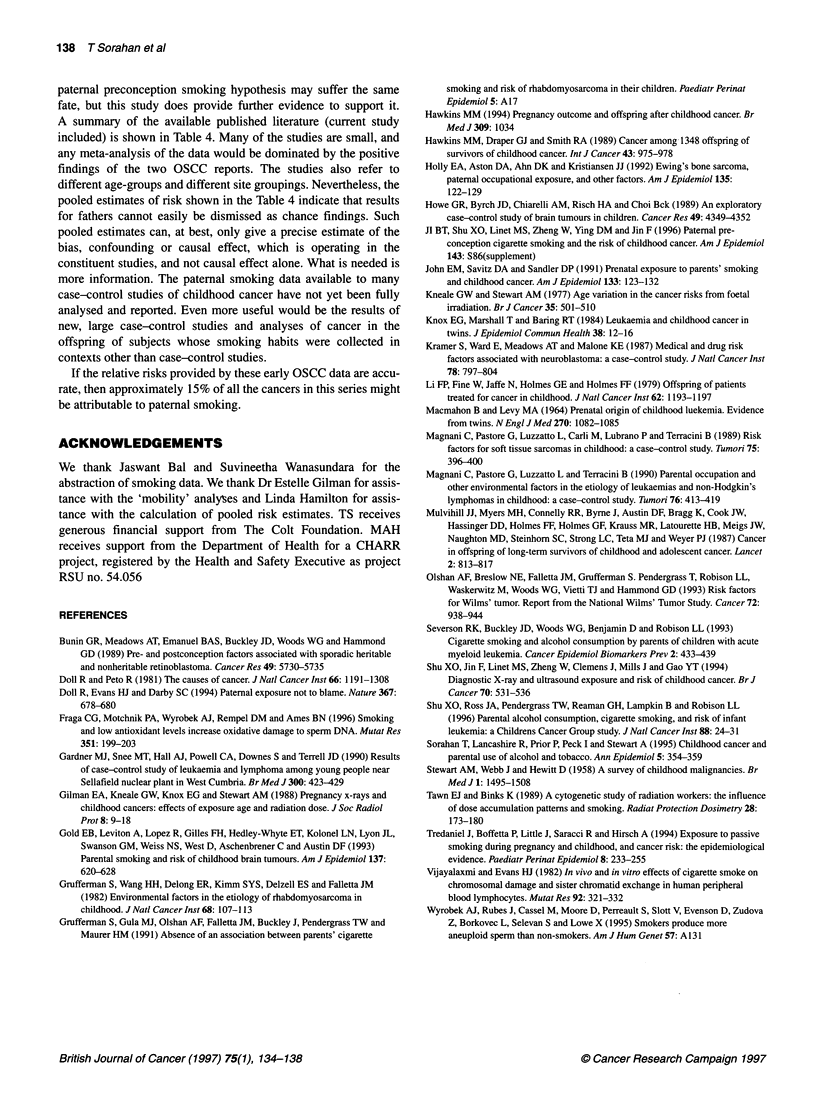# Childhood cancer and parental use of tobacco: deaths from 1953 to 1955.

**DOI:** 10.1038/bjc.1997.22

**Published:** 1997

**Authors:** T. Sorahan, R. J. Lancashire, M. A. HultÃ©n, I. Peck, A. M. Stewart

**Affiliations:** Institute of Occupational Health, University of Birmingham, Edgbaston, UK.

## Abstract

Parental smoking data have been abstracted from the interview records of the case-control study that first indicated that pregnancy radiographs are a cause of childhood cancer (Oxford Survey of Childhood Cancers, deaths from 1953 to 1955). Reported smoking habits for the parents of 1549 children who died from cancer were compared with similar information for the parents of 1549 healthy controls (matched pairs analysis). There was a statistically significant positive trend between paternal daily consumption of tobacco and the risk of childhood cancer (P< 0.001). This association could not be explained by maternal smoking, social class, paternal or maternal age at the birth of the survey child, sibship position or obstetric radiography. About 15% of all childhood cancers in this series could be attributable to paternal smoking.


British Journal of Cancer (1997) 75(1), 134–138
© 1997 Cancer Research Campaign

### Childhood cancer and parental use of tobacco: deaths from 1953 to 1955

**T Sorahan[1], RJ Lancashire[2], MA Hultén[3], I Peck[1] and AM Stewart[2]**

[1]Institute of Occupational Health and [2]Department of Public Health and Epidemiology, University of Birmingham, Edgbaston, Birmingham B15 2TT; [3]LSF Research Unit, Regional Genetic Laboratory and Consultancy Services, Birmingham Heartlands Hospital NHS Trust, Yardley Green Road, Birmingham B9 5PX, UK

**Summary** Parental smoking data have been abstracted from the interview records of the case–control study that first indicated that pregnancy radiographs are a cause of childhood cancer (Oxford Survey of Childhood Cancers, deaths from 1953 to 1955). Reported smoking habits for the parents of 1549 children who died from cancer were compared with similar information for the parents of 1549 healthy controls (matched pairs analysis). There was a statistically significant positive trend between paternal daily consumption of tobacco and the risk of childhood cancer ($P < 0.001$). This association could not be explained by maternal smoking, social class, paternal or maternal age at the birth of the survey child, sibship position or obstetric radiography. About 15% of all childhood cancers in this series could be attributable to paternal smoking.

**Keywords:** childhood cancer; smoking; case–control study

A recent review of the published literature on childhood cancer risks and parental use of tobacco concluded that 'the associations between maternal smoking during pregnancy and childhood cancer have been studied intensively, but there is no clear association overall, or for specific sites' (Tredaniel et al, 1994). The review also summarized information on paternal smoking from 13 case–control studies. Many of these studies were small in size and a total of only 1953 childhood cancers (varying diagnostic groups) formed the combined case series. The review concluded that '... no clear associations have been identified'.

A further four case–control study reports (additional combined series of 2772 cases) are now available, which include information on paternal smoking and childhood cancer risks (Severson et al, 1993; Shu et al, 1994, 1996; Sorahan et al, 1995). No association was found with maternal smoking in any of these reports but, in three of them, positive associations were found for paternal smoking (Shu et al, 1994, 1996; Sorahan et al, 1995). In the largest of these studies, reported consumptions of alcohol and tobacco for the parents of 1641 children who died from cancer in England and Wales during the period 1977–81 were compared with similar information for the parents of 1641 healthy control children (Sorahan et al, 1995). These data were obtained from the interview records of the Oxford Survey of Childhood Cancers (OSCC) and relations between maternal consumption of cigarettes and birth weights indicated that the (maternal) smoking data were equally reliable for case and control parents. For mothers, consumption of cigarettes was not shown to be associated with an increased risk of childhood cancer, whereas there was a statistically significant positive trend ($P < 0.001$) between daily consumption of cigarettes by fathers and childhood cancer risks. Earlier OSCC data have, therefore, been revisited to seek further information on this topic.

*Received 19 June 1996*
*Revised 3 September 1996*
*Accepted 13 September 1996*

*Correspondence to:* T Sorahan

The hypothesis to be examined was as follows: paternal cigarette smoking is a risk factor for the overall grouping of all childhood cancers and maternal cigarette smoking is unimportant.

### MATERIALS AND METHODS

The OSCC, a nationwide case–control study into the aetiology of childhood cancer, is one of the largest case–control studies in the history of medicine (Stewart et al, 1958; Gilman et al, 1988). The survey began in Oxford in 1955, but has been located at the University of Birmingham since 1975. The survey has sought to interview the parents (usually the mother) of all children dying of solid cancers, leukaemia or allied malignant conditions before their sixteenth birthday in England, Wales and Scotland for the period 1953–84. A number of standard questionnaires, covering a wide range of social and medical topics, have been used during the course of this prolonged study. Data on parental smoking habits are not available for all years of the study, but such information is available in the interview records relating to deaths from 1953 to 1955. Preliminary analyses of these data were published many years ago, but the amount of smoking was not considered (Stewart et al, 1958).

There were 1952 childhood cancer deaths in England, Wales and Scotland for the period 1953–55. Interview data had been obtained from the parents of 1631 (83.6%) of these children. Parents of 112 case children had refused to participate with the survey, a further group of 94 case parents had moved abroad or to an unknown address, and the remaining 115 case parents had not replied to survey requests, their general practitioner had advised the survey not to approach them, or arrangements to carry out interviews had fallen through. The response rate from case parents approached was thus 87.8% [1631/(1952–94)]. Some 16% of the interviewed case parents had moved local authority area between the birth and death of the survey child.

For each case child with interview data, a 'control list' of six children, matched for sex and date of birth, was selected from the birth register of the local authority area in which the case child died. Control parents were contacted in turn until one control

**Table 1** Relative risks of childhood cancers for parental smoking habits, deaths from 1953 to 1955, 1549 matched pairs

| Variable with levels | Cases | Controls | RR (95% CI) Separate analysis of variables | | Simultaneous analysis of variables | | Additional adjustments[a] | |
|---|---|---|---|---|---|---|---|---|
| Smoking habit of mother | | | | | | | | |
| Nil (<1 c.p.d.)[b] | 774 | 819 | 1.0 | | | | | |
| Slight (1–9 c.p.d.) | 442 | 451 | 1.04 | (0.88–1.22) | 1.01 | (0.85–1.19) | 0.99 | (0.83–1.18) |
| Moderate (10–20 c.p.d.) | 258 | 208 | 1.35** | (1.08–1.67) | 1.26* | (1.02–1.58) | 1.23 | (0.98–1.54) |
| Heavy (>20 c.p.d.) | 30 | 23 | 1.41 | (0.81–2.48) | 1.34 | (0.76–2.37) | 1.28 | (0.71–2.32) |
| NK | 45 | 48 | 0.99 | (0.65–1.53) | 0.63 | (0.29–1.36) | 0.65 | (0.28–1.48) |
| (*P* for trend)[c] | | | (0.013) | | (0.045) | | (0.092) | |
| Smoking habit of father | | | | | | | | |
| Nil (<1 c.p.d. or <1 p.p.d.)[d] | 263 | 302 | 1.0 | | | | | |
| Slight (1–9 c.p.d. or <3 o.p.w.)[e] | 356 | 409 | 0.99 | (0.80–1.24) | 1.01 | (0.81–1.26) | 1.03 | (0.81–1.29) |
| Moderate (10–20 c.p.d. or 3–6 o.p.w.) | 677 | 623 | 1.26* | (1.03–1.55) | 1.25* | (1.02–1.54) | 1.31* | (1.06–1.62) |
| Heavy (>20 c.p.d. or >6 o.p.w.) | 203 | 170 | 1.38* | (1.06–1.79) | 1.33* | (1.02–1.74) | 1.42* | (1.08–1.87) |
| NK | 50 | 45 | 1.28 | (0.83–1.97) | 1.89 | (0.89–4.03) | 1.89 | (0.84–4.24) |
| (*P* for trend)[c] | | | (<0.001) | | (0.003) | | (<0.001) | |

*$P<0.05$, **$P<0.01$. NK, not known. [a]Parental smoking habits analysed simultaneously with social class (five levels: I, II, III, IV, V), age of father at birth of survey child (five levels: <24, 25–29, 30–34, 35–39, >40 years), age of mother at birth of survey child (six levels: <20, 20–24, 25–29, 30–34, 35–39, >40 years), sibship position (five levels: 1, 2, 3, 4, >5) and obstetric radiography (yes/no). [b]c.p.d., cigarettes per day. [c]Ignoring NK category. [d]p.p.d., pipe per day. [e]o.p.w., ounces of pipe tobacco per week.

**Table 2** Relative risks of childhood cancers, by type of tumour, associated with smoking habits of parents, deaths from 1953 to 1955

| Type of tumour | Matched pairs | Smoking habit of mother[a] RR[b] | (95% CI) | Smoking habit of father[a] RR[b] | (95% CI) |
|---|---|---|---|---|---|
| Acute lymphatic leukaemia | 367 | 1.24* | (1.01–1.52) | 1.08 | (0.91–1.27) |
| Myeloid leukaemia | 115 | 1.20 | (0.85–1.68) | 0.98 | (0.73–1.32) |
| Monocytic leukaemia | 27 | 1.21 | (0.58–2.54) | 1.10 | (0.61–2.01) |
| Other and unspecified leukaemia | 216 | 1.18 | (0.91–1.55) | 1.14 | (0.93–1.39) |
| Lymphoma | 125 | 0.79 | (0.55–1.14) | 1.37* | (1.02–1.83) |
| Wilms' tumour | 133 | 0.98 | (0.71–1.36) | 1.01 | (0.77–1.34) |
| CNS cancers | 229 | 1.04 | (0.81–1.35) | 1.20 | (0.96–1.51) |
| Neuroblastoma | 138 | 0.93 | (0.68–1.28) | 1.48* | (1.09–2.02) |
| Bone cancers | 22 | 0.92 | (0.42–2.00) | 1.51 | (0.75–3.04) |
| Other solid cancers | 148 | 1.25 | (0.91–1.72) | 1.13 | (0.84–1.51) |
| Benign tumours | 29 | 1.70 | (0.68–4.24) | 0.60 | (0.28–1.27) |
| All diagnoses | 1549 | 1.11* | (1.00–1.22) | 1.13** | (1.05–1.23) |

*$P<0.05$, **$P<0.01$. [a]Only first four levels of smoking habit (see Table 1) are considered, i.e. nil, slight, moderate and heavy; levels are coded 1–4 and the variable is treated as a continuous variable. Maternal and paternal habits are analysed simultaneously. [b]These relative risks refer to a change of one level for smoking habit; a relative risk that is significantly different from unity indicates a statistically significant trend of risk with smoking habit.

family agreed to be interviewed. Interview data were obtained for 1622 control children (907 first choices, 342 second choices and 373 later choices). Only 56% of first choices may seem a low percentage but the birth registers from which the controls were selected had been compiled, on average, six or seven years before the survey began and about 25% of the required control families were found to have definitely left the district; only 6% of the control mothers approached refused to cooperate with the survey (Stewart et al, 1958). For some 94% of the 1622 matched pairs, the case and control parents within each pair were interviewed by the same person, usually a physician or nurse from the local health authority. For the remaining matched pairs, the case parents had moved locality between the death of the child and the time of the interview, and different interviewers were used for case and control parents.

For the purpose of this report, the interview folders of all matched pairs were reviewed and information on parental use of tobacco was abstracted and amalgamated with existing study computer files. The interview questionnaire requested responses in terms of 'nil', 'slight', 'moderate', or 'heavy' and the definitions of these terms as supplied to the interviewers are summarized in Table 1. The question was directed at current rather than past smoking habits, and for fathers, responses could refer to use of cigarettes or pipe tobacco. After excluding 20 matched pairs in which the case child was adopted and 53 matched pairs in which smoking information was not sought, case and control data relating to tobacco consumption (1549 matched pairs) were compared (with and without adjustment for other variables) by means of (multiple) conditional logistic regression using the EGRET program. The odds ratio was used to obtain estimates of

© Cancer Research Campaign 1997

**Table 3** Relative risks of childhood cancers by smoking habits of one or both parents

| Variable with levels | Cases | Controls | RR | (95% CI) | RR | (95% CI) |
|---|---|---|---|---|---|---|
| | | | | | Additional adjustments[a] | |
| Moderate or heavy smoker | | | | | | |
| Neither parent | 550 | 648 | 1.0 | | | |
| Mother only | 66 | 63 | 1.27 | (0.88–1.82) | 1.21 | (0.84–1.75) |
| Father only | 655 | 618 | 1.27** | (1.08–1.50) | 1.30** | (1.10–1.53) |
| Both parents | 220 | 165 | 1.66*** | (1.30–2.12) | 1.70*** | (1.32–2.18) |
| NK | 58 | 55 | 1.27 | (0.85–1.88) | 1.21 | (0.80–1.81) |

*$P$<0.05, **$P$<0.01, ***$P$<0.001. NK, not known. See footnote a in Table 1.

**Table 4** Summary of published studies providing estimates of childhood cancer risks in relation to paternal smoking

| Reference | No. of cases | Type of cancer | Smoking habit[a] | Paternal smoking[b] RR | (95% CI) | Maternal smoking[b] RR | (95% CI) |
|---|---|---|---|---|---|---|---|
| Grufferman et al (1982) | 33 | Rhabdomyosarcomas | | 3.9 | (1.5–9.6) | 0.8 | (0.3–2.0) |
| Kramer et al (1987)[c] | 104 | Neuroblastomas | | 1.30 | (0.75–2.24) | 1.26 | (0.69–2.31) |
| Bunin et al (1989) | 115 | Non-heritable retinoblastomas | | 1.2 | (0.7–2.3) | 1.1 | (0.6–2.1) |
| | 67 | Sporadic heritable retinoblastomas | | 2.3 | (0.8–7.0) | 2.0 | (0.7–6.5) |
| Howe et al (1989) | 74 | Brain tumours | | 1.13 | (0.62–2.09) | 1.42 | (0.70–3.00) |
| Magnani et al (1989) | 52 | Soft-tissue sarcomas | 1–15 | 1.0 | (0.4–2.4) | 0.7 | (0.3–1.4) |
| | | | ≥16 | 0.8 | (0.4–2.0) | 0.0[d] | |
| Magnani et al (1990) | 142 | Acute lymphocytic leukaemia | | 0.9 | (0.6–1.5) | 0.7 | (0.5–1.1) |
| | 22 | Other acute leukaemia | | 0.9 | (0.3–2.1) | 2.0 | (0.8–4.8) |
| | 19 | Non-Hodgkin's lymphoma | | 6.7 | (1.0–43.4) | 1.7 | (0.7–4.5) |
| Grufferman et al (1991) | 322 | Rhabdomyosarcomas | | 1.0 | (0.7–1.4) | 1.0 | (0.8–1.4) |
| John et al (1991) | 233 | All sites | 1–10 | 1.9 | (0.9–3.9) | 1.3 | (0.7–2.4) |
| | | | 11–20 | 1.3 | (0.8–2.1) | 1.3 | (0.8–2.2) |
| | | | ≥21 | 1.0 | (0.6–1.8) | | |
| Holly et al (1992) | 43 | Ewing's sarcoma | | 0.9 | (0.4–1.9) | 1.1 | (0.5–2.4) |
| Gold et al (1993) | 361 | Brain tumours | 1–19 | 0.68 | (0.39–1.19) | 0.84 | (0.56–1.27) |
| | | | ≥20 | 1.07 | (0.79–1.45) | 1.00 | (0.70–1.43) |
| Olshan et al (1993) | 200 | Wilms' tumours | 1–9 | 0.47 | (0.14–1.60) | 0.79 | (0.35–1.81) |
| | | | ≥10 | 1.11 | (0.69–1.78) | 0.73 | (0.40–1.34) |
| Ji et al (1994)[e] | 642 | All sites | 1–5 py | 1.3 | (0.9–1.7) | | |
| | | | ≥5 py | 1.7 | (1.2–2.5) | | |
| Sorahan et al (1995) | 1641 | All sites | 1–9 | 1.20 | (0.81–1.78) | 0.98 | (0.73–1.30) |
| | | | 10–19 | 1.24 | (0.98–1.56) | 1.18 | (0.96–1.44) |
| | | | 20–29 | 1.26 | (1.05–1.50) | 0.98 | (0.80–1.21) |
| | | | 30–39 | 1.35 | (1.03–1.78) | 0.90 | (0.56–1.46) |
| | | | ≥40 | 1.47 | (1.07–2.01) | 1.60 | (0.87–2.96) |
| Shu et al (1996) | 302 | Leukaemias | 1–10 | 1.39 | (0.69–2.82) | 0.71 | (0.51–1.01) |
| | | | 11–20 | 1.15 | (0.74–1.80) | | |
| | | | >20 | 1.36 | (0.81–2.28) | | |
| This report[f] | 1549 | All sites | 1–9 | 1.03 | (0.81–1.29) | 0.99 | (0.83–1.18) |
| | | | 10–20 | 1.31 | (1.06–1.62) | 1.23 | (0.98–1.54) |
| | | | >20 | 1.42 | (1.08–1.87) | 1.28 | (0.71–2.32) |
| Pooled estimate (smoker *vs* non-smoker)[g] | | | | 1.23 | (1.14–1.33) | 1.05 | (0.96–1.14) |

[a]Unspecified units are in cigarettes per day, e.g. 1–15 = 1–15 c.p.d; py = pack–years of smoking, e.g. 1–5 py = 1–5 pack–years of smoking (pack =20 cigarettes); unspecified smoking habit (blank entry) refers to smoker/non-smoker comparisons. [b]Risks relative to non-smokers. If the paper includes information for a number of smoking variables, results for smoking before the relevant pregnancy are selected. [c]Confidence intervals calculated by current authors. [d]Not included in pooled estimate. [e]Additional information supplied by original authors. [f]From simultaneous analysis of parental smoking habits. [g]Pooled estimate calculated from a weighted average of the log-relative risks, with inverse variance weights. The confidence interval for the pooled log-relative risk calculated from its standard error, given approximately ns $\sqrt{N/\Sigma SE_i}$, where $N$ is the number of odds ratios to be pooled and $i$ = 1,2 ... $N$.

relative risk (RR). Risks are shown relative to a baseline risk of 1.0 for the 'nil' category (essentially non-smokers).

### RESULTS

Relative risks for all types of childhood cancers combined are shown by parental smoking habits in Table 1. Smoking habits of mothers and fathers are first analysed separately (two analyses), then adjusting for the other parent's habits (one further analysis), and then adjusting not only for the other parent's habits, but also for their ages at the birth of the survey child, social class (based on occupation of father), sibship position and obstetric radiography (one further analysis). The purpose of the simultaneous analyses was to allow for the effects of other variables, so that the

© Cancer Research Campaign 1997

independent effects of each variable can be examined. Relative risks for moderate and heavy smoking by mothers were reduced by the inclusion of potential confounding variables, whereas those for moderate and heavy smoking by fathers were increased. None of the point estimates of relative risk shown for mothers in the final columns of Table 1 was statistically significant, whereas relative risks for both moderate and heavy smoking by fathers were statistically significant ($P < 0.05$). In addition, the positive trend between amount of maternal smoking (four levels, nil to heavy) and childhood cancer risk was not statistically significant ($P = 0.09$), whereas the corresponding trend for paternal smoking was highly significant ($P < 0.001$).

The analysis summarized in the final column of Table 1 was repeated for those 1291 matched pairs in which the case parents (and by definition, the control parents) had not moved local authority area between the birth and death of the survey child. Similar results were obtained (point estimates of relative risks for paternal smoking habit were as follows: slight, 1.02; moderate, 1.25; heavy, 1.55).

Relative risks associated with paternal and maternal daily smoking habits are shown for ten diagnostic groups in Table 2. To enable a summary to be given in a single table, relative risks for a change of one smoking level are provided. All confidence intervals for the site-specific relative risks include the corresponding point estimate of relative risk for all types of childhood cancers combined.

The role of interactions between maternal and paternal habits is examined in Table 3, which shows moderate or heavy use by neither parent, mother only, father only, and both parents, with and without the adjustments described above. Statistically significant risks are shown for father only and for both parents; the point estimate of relative risk for both parents (1.70) was much higher than for father only (1.30).

### DISCUSSION

The study provides more convincing evidence of an effect from paternal smoking than from maternal smoking though very few mothers (66 cases and 63 controls) reported 'moderate' or 'heavy' smoking in the absence of the same claim for the relevant fathers.

The majority of childhood cancers have prenatal origins (MacMahon and Levy, 1964; Kneale and Stewart, 1977), and analyses of childhood cancer in twins suggest that determining events of these diseases have to be experienced both by the zygote (or the germ cells giving rise to the zygote) and by the fetus (or child) (Knox et al, 1984). It follows that, if the observed smoking association is causal in nature, this might be caused either by the effects of smoking on sperm or the effects of passive smoking on young infants, or both. However, any effect of passive smoking would tend to involve mothers more than fathers and other studies have shown that maternal smoking is unlikely to be an important risk factor (Severson et al, 1993; Tredaniel et al, 1994; Shu et al, 1994, 1996; Sorahan et al, 1995).

A role for paternal preconceptional smoking on childhood cancer risks is biologically plausible and there is no reason to believe that such a risk factor would only apply to certain types of childhood cancer. Indeed, a variety of genetic diseases in newborns might also be expected. It is well known that urine of smokers contains carcinogenic substances and that smoking per se is associated with an increased risk of malignancy (Doll and Peto, 1981). It is further well established that blood lymphocytes of smokers show significantly increased chromosome damage as evaluated by molecular cytogenetics technology (Vijayalaxmi and Evans, 1982; Tawn and Binks, 1989). In addition, it has been demonstrated recently that smokers have raised levels of chromosome aberrations in sperm (Wyrobek et al, 1995) and that smokers have increased oxidative damage in sperm DNA (Fraga et al, 1996). If paternal smoking is affecting childhood cancer risks via the production of mutated sperms, a hereditary risk ought to carry through to the offspring of the survivors of childhood cancer. The expression of this risk would clearly depend on the mode of genetic transmission and the penetrance of the defective gene. The three larger cohort studies of cancer in the offspring of survivors of childhood cancer together comprise about 4000 offspring (Li et al, 1979; Mulvihill et al, 1987; Hawkins et al, 1989;) but provide little suggestion of hereditary risks. In these studies, 'when inherited retinoblastomas and a family with Sipple's syndrome were excluded there were only seven cases [of cancer in the offspring]. About five cases would have been expected ...' (Hawkins 1994).

The point estimates of site-specific risks provided by the earlier OSCC findings (Sorahan et al, 1995) are quite different to those contained in the present report. However, if paternal smoking has a discernible risk on the childhood cancers overall, then inconsistency is not a problem; the variation in the ranking of site-specific risks from study to study may represent no more than chance fluctuations. It does not follow, of course, that each and every type of childhood cancer is necessarily affected by paternal smoking.

One key issue in evaluating the importance of these findings is the reliability of OSCC data. Unfortunately, data on birth weights of the case and control children were not available, and consequently, no independent check on the likely reliability of the maternal smoking data was possible. For the fathers' smoking habits there is no comparable test. The interviews were carried out in the period 1955–58, a period well before concerns about the health effects of smoking became a media topic and 'stop smoking' health campaigns were commonplace. Consequently, there was probably little difference between paternal smoking habits at the time of the interviews and earlier habits, and the reported smoking habits are likely to be more reliable than those available to more recent studies. The possibility of differential reporting between case and control parents remains. Other issues also need to be considered. The study achieved a very good response rate (for case–control studies), but the effects of having to ignore the non-responders are not known. The method of selecting controls means that 'mobile' families would tend to be under-represented in the control series and it is possible that 'mobile' fathers smoke more than the average. Analyses restricted to 'non-mobile' cases however, produced similar findings. It seems unlikely that the inclusion of childhood cancer survivors would have led to materially different results for two reasons. Firstly, in the 1950s, only a small percentage of children diagnosed with cancer before the age of 16 years survived past their sixteenth birthday; and secondly, it seems unlikely that the findings for paternal smoking could be an artifact caused by paternal smoking increasing cancer mortality rates in children diagnosed with cancer.

Caution is required. There is no convincing example of an environmentally induced paternal preconception risk factor for childhood cancer. In this area of research, paternal preconception exposure to external ionizing radiation provides a recent example of an initial finding of potential importance (Gardner et al, 1990) that failed to be replicated in later studies (Doll et al, 1994). The

© Cancer Research Campaign 1997

paternal preconception smoking hypothesis may suffer the same fate, but this study does provide further evidence to support it. A summary of the available published literature (current study included) is shown in Table 4. Many of the studies are small, and any meta-analysis of the data would be dominated by the positive findings of the two OSCC reports. The studies also refer to different age-groups and different site groupings. Nevertheless, the pooled estimates of risk shown in the Table 4 indicate that results for fathers cannot easily be dismissed as chance findings. Such pooled estimates can, at best, only give a precise estimate of the bias, confounding or causal effect, which is operating in the constituent studies, and not causal effect alone. What is needed is more information. The paternal smoking data available to many case–control studies of childhood cancer have not yet been fully analysed and reported. Even more useful would be the results of new, large case–control studies and analyses of cancer in the offspring of subjects whose smoking habits were collected in contexts other than case–control studies.

If the relative risks provided by these early OSCC data are accurate, then approximately 15% of all the cancers in this series might be attributable to paternal smoking.

### ACKNOWLEDGEMENTS

We thank Jaswant Bal and Suvineetha Wanasundara for the abstraction of smoking data. We thank Dr Estelle Gilman for assistance with the 'mobility' analyses and Linda Hamilton for assistance with the calculation of pooled risk estimates. TS receives generous financial support from The Colt Foundation. MAH receives support from the Department of Health for a CHARR project, registered by the Health and Safety Executive as project RSU no. 54.056

### REFERENCES

Bunin GR, Meadows AT, Emanuel BAS, Buckley JD, Woods WG and Hammond GD (1989) Pre- and postconception factors associated with sporadic heritable and nonheritable retinoblastoma. *Cancer Res* **49**: 5730–5735

Doll R and Peto R (1981) The causes of cancer. *J Natl Cancer Inst* **66**: 1191–1308

Doll R, Evans HJ and Darby SC (1994) Paternal exposure not to blame. *Nature* **367**: 678–680

Fraga CG, Motchnik PA, Wyrobek AJ, Rempel DM and Ames BN (1996) Smoking and low antioxidant levels increase oxidative damage to sperm DNA. *Mutat Res* **351**: 199–203

Gardner MJ, Snee MT, Hall AJ, Powell CA, Downes S and Terrell JD (1990) Results of case–control study of leukaemia and lymphoma among young people near Sellafield nuclear plant in West Cumbria. *Br Med J* **300**: 423–429

Gilman EA, Kneale GW, Knox EG and Stewart AM (1988) Pregnancy x-rays and childhood cancers: effects of exposure age and radiation dose. *J Soc Radiol Prot* **8**: 9–18

Gold EB, Leviton A, Lopez R, Gilles FH, Hedley-Whyte ET, Kolonel LN, Lyon JL, Swanson GM, Weiss NS, West D, Aschenbrener C and Austin DF (1993) Parental smoking and risk of childhood brain tumours. *Am J Epidemiol* **137**: 620–628

Grufferman S, Wang HH, Delong ER, Kimm SYS, Delzell ES and Falletta JM (1982) Environmental factors in the etiology of rhabdomyosarcoma in childhood. *J Natl Cancer Inst* **68**: 107–113

Grufferman S, Gula MJ, Olshan AF, Falletta JM, Buckley J, Pendergrass TW and Maurer HM (1991) Absence of an association between parents' cigarette smoking and risk of rhabdomyosarcoma in their children. *Paediatr Perinat Epidemiol* **5**: A17

Hawkins MM (1994) Pregnancy outcome and offspring after childhood cancer. *Br Med J* **309**: 1034

Hawkins MM, Draper GJ and Smith RA (1989) Cancer among 1348 offspring of survivors of childhood cancer. *Int J Cancer* **43**: 975–978

Holly EA, Aston DA, Ahn DK and Kristiansen JJ (1992) Ewing's bone sarcoma, paternal occupational exposure, and other factors. *Am J Epidemiol* **135**: 122–129

Howe GR, Byrch JD, Chiarelli AM, Risch HA and Choi Bck (1989) An exploratory case–control study of brain tumours in children. *Cancer Res* **49**: 4349–4352

JI BT, Shu XO, Linet MS, Zheng W, Ying DM and Jin F (1996) Paternal preconception cigarette smoking and the risk of childhood cancer. *Am J Epidemiol* **143**: S86(supplement)

John EM, Savitz DA and Sandler DP (1991) Prenatal exposure to parents' smoking and childhood cancer. *Am J Epidemiol* **133**: 123–132

Kneale GW and Stewart AM (1977) Age variation in the cancer risks from foetal irradiation. *Br J Cancer* **35**: 501–510

Knox EG, Marshall T and Baring RT (1984) Leukaemia and childhood cancer in twins. *J Epidemiol Commun Health* **38**: 12–16

Kramer S, Ward E, Meadows AT and Malone KE (1987) Medical and drug risk factors associated with neuroblastoma: a case–control study. *J Natl Cancer Inst* **78**: 797–804

Li FP, Fine W, Jaffe N, Holmes GE and Holmes FF (1979) Offspring of patients treated for cancer in childhood. *J Natl Cancer Inst* **62**: 1193–1197

Macmahon B and Levy MA (1964) Prenatal origin of childhood luekemia. Evidence from twins. *N Engl J Med* **270**: 1082–1085

Magnani C, Pastore G, Luzzatto L, Carli M, Lubrano P and Terracini B (1989) Risk factors for soft tissue sarcomas in childhood: a case–control study. *Tumori* **75**: 396–400

Magnani C, Pastore G, Luzzatto L and Terracini B (1990) Parental occupation and other environmental factors in the etiology of leukaemias and non-Hodgkin's lymphomas in childhood: a case–control study. *Tumori* **76**: 413–419

Mulvihill JJ, Myers MH, Connelly RR, Byrne J, Austin DF, Bragg K, Cook JW, Hassinger DD, Holmes FF, Holmes GF, Krauss MR, Latourette HB, Meigs JW, Naughton MD, Steinhorn SC, Strong LC, Teta MJ and Weyer PJ (1987) Cancer in offspring of long-term survivors of childhood and adolescent cancer. *Lancet* **2**: 813–817

Olshan AF, Breslow NE, Falletta JM, Grufferman S. Pendergrass T, Robison LL, Waskerwitz M, Woods WG, Vietti TJ and Hammond GD (1993) Risk factors for Wilms' tumor. Report from the National Wilms' Tumor Study. *Cancer* **72**: 938–944

Severson RK, Buckley JD, Woods WG, Benjamin D and Robison LL (1993) Cigarette smoking and alcohol consumption by parents of children with acute myeloid leukemia. *Cancer Epidemiol Biomarkers Prev* **2**: 433–439

Shu XO, Jin F, Linet MS, Zheng W, Clemens J, Mills J and Gao YT (1994) Diagnostic X-ray and ultrasound exposure and risk of childhood cancer. *Br J Cancer* **70**: 531–536

Shu XO, Ross JA, Pendergrass TW, Reaman GH, Lampkin B and Robison LL (1996) Parental alcohol consumption, cigarette smoking, and risk of infant leukemia: a Childrens Cancer Group study. *J Natl Cancer Inst* **88**: 24–31

Sorahan T, Lancashire R, Prior P, Peck I and Stewart A (1995) Childhood cancer and parental use of alcohol and tobacco. *Ann Epidemiol* **5**: 354–359

Stewart AM, Webb J and Hewitt D (1958) A survey of childhood malignancies. *Br Med J* **1**: 1495–1508

Tawn EJ and Binks K (1989) A cytogenetic study of radiation workers: the influence of dose accumulation patterns and smoking. *Radiat Protection Dosimetry* **28**: 173–180

Tredaniel J, Boffetta P, Little J, Saracci R and Hirsch A (1994) Exposure to passive smoking during pregnancy and childhood, and cancer risk: the epidemiological evidence. *Paediatr Perinat Epidemiol* **8**: 233–255

Vijayalaxmi and Evans HJ (1982) *In vivo* and *in vitro* effects of cigarette smoke on chromosomal damage and sister chromatid exchange in human peripheral blood lymphocytes. *Mutat Res* **92**: 321–332

Wyrobek AJ, Rubes J, Cassel M, Moore D, Perreault S, Slott V, Evenson D, Zudova Z, Borkovec L, Selevan S and Lowe X (1995) Smokers produce more aneuploid sperm than non-smokers. *Am J Hum Genet* **57**: A131

© Cancer Research Campaign 1997